# Perioperative complications and postoperative outcome of primary total knee arthroplasty in octogenarians – A systematic review

**DOI:** 10.1016/j.jor.2025.05.025

**Published:** 2025-05-20

**Authors:** Annemarie Rusche, Georg Osterhoff, Andreas Roth, Nikolas Schopow

**Affiliations:** Department for Orthopedics, Trauma Surgery and Plastic Surgery, University Hospital Leipzig, Leipzig, Germany

## Abstract

Osteoarthritis is a common disease worldwide, most commonly affecting the knee. With the rise of obesity, paired with higher life expectancy, prevalence of osteoarthritis of the knee will rise as well. Therefore, Total Knee Arthroplasty (TKA) rates are expected to increase 85 %, estimating 1.26 million procedures worldwide in 2030. Patients will become older with demographic changes and more elderly people will be eligible for procedure, a patient group is often considered vulnerable, with a higher risk of mortality and complications. This systematic review aimed to compare perioperative outcomes after TKA in Octogenarians with those in a younger cohort. After screening 33,336 publications on total joint arthroplasty in the elderly in the PubMed and Web of Science databases, four studies investigating TKA and matching criteria were included. No notable variations in outcomes were detected among elderly compared to younger patients. Eligibility for a procedure should be based on comorbidities and overall health status rather than age alone.

## 1 Introduction

Osteoarthritis (OA) is a significant burden on healthcare systems globally. With an aging population and high BMI as critical risk factors, the impact of OA will become even greater. The knee is the most affected joint, with^5^ an estimated 642 million people projected to have knee OA by 2050.^6^ Osteoarthritis (OA) is characterized by the gradual deterioration of articular cartilage and subsequent inflammation within the synovial cavity. The origin of the disease is multi etiological, involving individual risk factors and past injuries. Clinically, OA is characterized by chronic pain, joint stiffness and swelling.^33^

Total Knee Arthroplasty (TKA) is the most common treatment for end-stage OA. In 2021, 119 per 100.000 inhabitants were performed.^20^ With rising prevalence for OA, the need for TKA will rise as well. These epidemiologic changes lead to a larger group of elderly patient eligible for procedure.

Given the high frequency of this procedure, it is crucial to investigate its consequences, especially in older individuals. Octogenarians, those aged over 80 years, are often seen as a fragile patient group. But does advancing age increase mortality after TKA in elderly? Is age a significant risk factor for longer hospital stay duration? Do Octogenarians experience more perioperative complications and if so, which? And which improvements are necessary to reduce possible complications?

This systematic review aimed to compare the perioperative outcomes of TKA performed on Octogenarians with those of younger control groups. We sought to identify risk factors that can help determine patient eligibility for these procedures, using more objective, clinically assessed parameters rather than age alone.

### 1.1 Search strategy and criteria

**Fig. 1:**
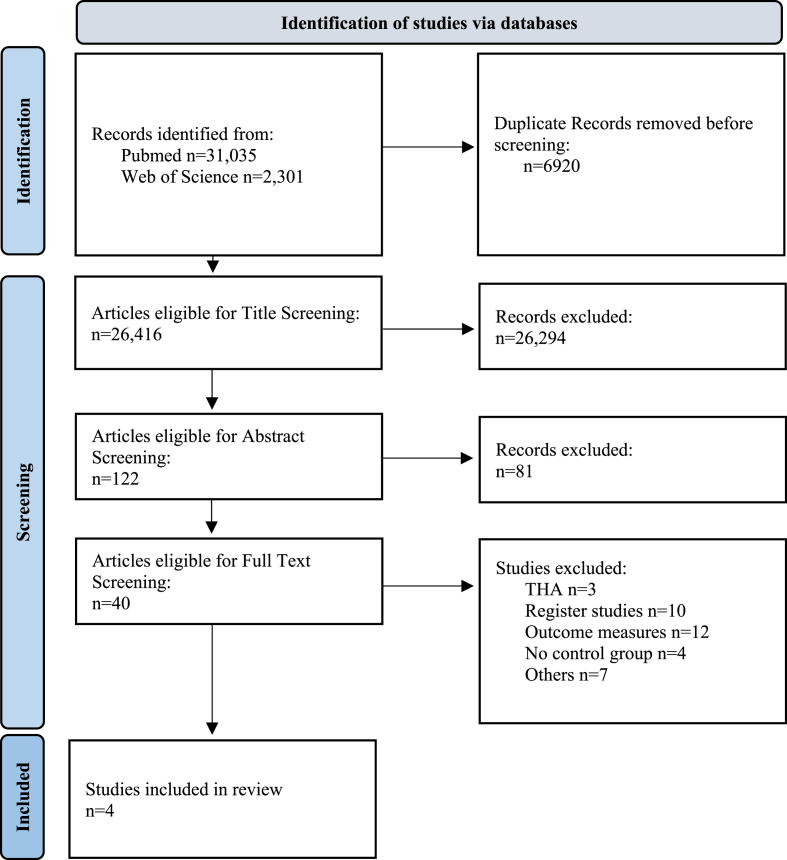
PRISMA flow diagram for systematic reviews The flow chart provides further details about the screening process. Identification of studies eligible for this systematic review followed PRISMA guidelines. Other reasons for exclusion were e.g. study design, cohort selection and research including TKA and THA simultaneously. TKA: Total Knee Arthroplasty. THA: Total Hip Arthroplasty.

This systematic review was registered with PROSPERO (CRD42022337168). Following the guidelines provided by the Cochrane Handbook for Systematic Reviews of Interventions and the PRISMA (Preferred Reporting Items for Systematic Reviews and Meta-Analyses) statement.^7^^,^^22^ We searched two different databases, PubMed and Web of Science. All publications were last searched August 23rd, 2022. The following search term was used: ((Total hip arthroplasty OR THA) OR (total knee arthroplasty OR TKA)) AND (geriatric OR elderly OR octogenarian)). Filters, including language (English, German, and French) and publication date (January 1, 2012, to May 31, 2024), were applied to refine the database search. Studies were researched and identified by two reviewers. For this systematic review the following including and excluding criteria were defined beforehand using the PICOS (patient, intervention, control, outcome, study) framework. Octogenarians had to be at least 80 years and undergo primary TKA. A younger control group was necessary. Studies with the following outcomes were included: mortality, Length of Stay (LoS), surgical and medical complications. Randomized controlled trials (RCT) and clinical trials were included. Publications without available full text, case reports, conference or congress communications, systematic reviews, and meta-analyses were not considered eligible. Non-primary procedures e.g., after trauma or revision surgery, and studies with less than 50 patients were excluded. 33,336 publications were found on both databases. Information about the search strategy is shown in the PRISMA 2020 flow diagram (Fig. 1). Data was exported from PubMed and Web of Science and further analyzed in Zotero and Excel. To minimize bias due to cohort size mismatch, registry studies were excluded. The main outcomes, mortality and LoS, were evaluated for the quality of certainty of evidence according to the Grading of Recommendations Assessment, Development and Evaluation (GRADE) criteria and for the risk of bias using the Risk of Bias In Non-Randomized Studies of Interventions (ROBINS-I) guidelines.^7^^,^^26^, ^28^

The Oxford Center for Evidence-Based Medicine (OCEBM) 2011 criteria were used to assess the level of evidence.^19^ Requests for supplementary data were made. Unfortunately, no additional data could be provided. References cited in included publications were screened additionally.

## 2 Results

### 2.1 General information

A total of 33,336 publications were identified based on the predefined criteria in both databases. After removing 6920 duplicates, 26,416 publications underwent title screening. Abstract screening was then conducted on 122 publications, narrowing it down to 40. After full-text screening and excluding registry studies four retrospective cohort studies were eligible, seen in Fig. 1.

### 2.2 Total knee arthroplasty

In total 5447 patients, 2233 Octogenarians and 3314 younger persons, undergoing TKA were analyzed in this systematic review.

In the study conducted by Kodaira et al. cohorts were not matched.^11^ Andreozzi et al. used a 1:1 matched pair approach, matching for BMI, follow-up duration and gender.^2^ Maempel et al. included the largest number of patients and outcomes were adjusted for different possible confounders amongst age.^17^ Kuo et al. matched 1:1 e.g. for gender, BMI and ASA score.^14^ Further information is provided in Table 1.

**Table 1:** – Total knee arthroplasty: Characteristics.

	Data type	Mean age in years (±SD)			Female gender in %			Mean BMI (±SD)			D-CCI		
		OC	YC	p	OC	YC	p	OC	YC	p	OC	YC	p
Kodaira (2019)∗	SC	82 (N/A)	71 (N/A)	<0.05	77	81	<0.05	25.1 (N/A)	27.0 (N/A)	<0.05	N/A	N/A	N/A
Andreozzi (2019)	SC	83 ± 3	64 ± 6	<0.001	70	70	1.0	27.1 ± 3.7	26.6 ± 2.6	0.33	N/A	N/A	0.21
Maempel (2015)∗∗	MC	83 (N/A)	68.67 (N/A)	N/A	N/A	N/A	N/A	N/A	N/A	N/A	N/A	N/A	N/A
Kuo (2014)	SC	82 ± 2.8	72 ± 2.2	<0.001	72	82	0.08	26.2 ± 3.0	29.2 ± 3.7	0.14	N/A	N/A	N/A

The table outlines study and patient characteristics, detailing the use of single-center (SC) and multi-center (MC) databases. Maempel et al. involved 5 institutions. Mean age and proportion of female patients within cohorts are presented. Body Mass Index (BMI) and Deyo-Charlson Comorbidity Index (D-CCI) provide further description about the Octogenarian cohort (OC) and the younger cohort (YC). p: statistical significance; SD: standard deviation; N/A: data not available. ∗No further information regarding p-value. ∗∗Cohorts were divided in three age groups, patients aged 75 or less and those aged between 75 and 80 years were summated.

Mortality rates after TKA vary from 0 % to 2 %, showing similar results to those after THA.^23^ No significant differences can be observed throughout age groups. Octogenarians had a longer hospital stay compared to their younger counterparts across all studies. Age does not seem to be a clear indicator for neither medical nor surgical complication rates. More detailed information about perioperative complications is presented in Table 2.

**Table 2:** – Total knee arthroplasty: Mortality, length of stay and complications.

	Mortality rate in %			Mean LoS in days (±sd)			Complications n (%)							
	OC	YC	p	OC	YC	p	OC	YC	p	Surgical complications	OC	YC	p	Medical complications
Kodaira (2019)	N/A	N/A	N/A	18.8 ± 0.3	16.8 ± 0.2	N/A	5	7	N/A	Loosening of implants, Periprosthetic joint infection	145	27	N/A	Confusion∗∗, delayed wound healing, bedsore
Andreozzi (2019)	2	0	0.498	5.8 ± 3.7	4.1 ± 2.3	<0.001	8 (8 %)	10 (10 %)	0.80	Not specified	N/A	N/A	N/A	Transfusion∗∗, Cardiac complications, Delirium
Maempel (2015)∗	0.86	0.36	N/A	N/A	N/A	N/A	2 %	3.3 %	N/A	Deep infection, revision, manipulation under anesthesia	75.4 %	32.46 %	N/A	Urinary catherization, Transfusion∗∗, lower respiratory tract infection
Kuo (2014)	0	0	N/A	6.1 ± 1.3	5.7 ± 1.2	0.061	4	1	0.20	Superficial wound infection	N/A	N/A	N/A	Anemia, transfusion∗∗, confusion∗∗

Primary outcomes for the Octogenarian cohort (OC) and the younger cohort (YC) are presented. Mortality rates were assessed within 90 days after procedure, with exception of Maempel et al., where mortality rates were assessed after one month. Three most common surgical and medical complications are listed. LoS: length of stay; N/A: data not available; SD: standard deviation; p: statistical significance. ∗Cohorts were divided in three age groups, patients aged 75 or less and those aged between 75 and 80 years were summated.∗∗ Statistically significant difference between cohorts.

Each study was evaluated in regard of certainty of evidence, presented in Table S1. According to GRADE criteria, evidence must be ranked “very low”.

## 3 Discussion

TKA is a frequently performed surgery, and the numbers are expected to rise in the coming years. With high prevalence of this surgery the importance of understanding mortality rates and perioperative outcomes after TKA in the elderly is heightened. The aim of this systematic review was to investigate the influence of advanced age on perioperative outcomes after TKA. Our findings indicate that TKA can be safely performed in Octogenarians if they are eligible for the procedure. Age alone should not be a criterion for exclusion.

### 3.1 Does advanced age increase mortality after TKA?

In previous literature, higher age was often associated with higher mortality rates.^4^^,^^10^

Based on the studies examined, no evidence of age-related excess mortality was found. Data were very heterogeneous. Maempel et al. found that mortality rates were highest in the cohort aged between 75 and 80, not in the cohort with patients aged over 80 years.^17^ This was potentially due to a preclinical selection in the oldest group, whilst frailty and lack of physical reserve are underestimated in the middle group.

### 3.2 Do elderly experience longer hospital stay duration?

Aligning with current literature, all Octogenarian cohorts had longer hospital stays than their younger counterparts.^2^^,^^4^^,^^11^^,^^14^^,^^17^ Age does not seem to be the only relevant variable. Frailty, e.g. measured in Hospital Frailty Risk Score (HFRS), is associated with longer duration of hospital stay as well and had a stronger impact on LoS than age.^15^

There are additional factors that may influence the duration of hospital stays. Kodaira et al. reported a notably longer hospital stay compared to the other studies included. This difference is likely due to their discharge criterion, which required patients to be able to walk without assistance, effectively integrating a portion of the rehabilitation process into the hospital stay.^11^ Cultural differences might also play a role, as longer lengths of stay have been frequently reported in studies conducted in Japan.^18^^,^^31^

### 3.3 Do Octogenarians experience more perioperative complications than their younger counterparts?

The heterogenic data revealed no higher frequency of short-term surgical complications among Octogenarians compared to younger patients, as shown in Table 2. Infection appeared in both groups with no significant difference.^11^^,^^17^ Implant loosening was even more frequent in the YC.^11^ The authors suspected higher level of physical activity in younger patients as a reasoning for aseptic loosening. In a recent meta-analysis, high physical activity level was neither associated with revision due to aseptic loosening nor all-cause revision, though.^12^ It is noticeable that Kodaira's YC weighs significantly more.^11^ Overweight and obese patient experience more aseptic loosening compared to patients at a normal bodyweight.^24^

Cardiovascular events occurred in each cohort despite age. Andreozzi et al. found that high comorbidity index rather than age was at greater risk for cardiac complications and thromboembolic events.^2^ Kodaira et al. observed significantly more cases of acute heart failure among elderly, but preoperative prevalence of ischemic heart disease was also higher among the OC. The history of heart disease is an independent risk factor for acute heart failure in patients with hip fractures.^30^

Elderly patients received significantly more blood transfusion.^2^^,^^14^^,^^17^ Postoperative confusion and delirium were also more frequent among the OC. [2; 11; 14.]

### 3.4 Which improvements could possibly reduce postoperative complications in octogenarians?

The prevalence of anemia is higher with rising age, which may result in lower preoperative hemoglobin (Hb).^27^ The change in Hb concentration after procedure is similar, 2.3 g/dl in the OC vs. 2.2 g/dl in the YC.^17^ But when preoperative Hb is already lower in elderly patients and considering earlier transfusion triggers, the likelihood of requiring a transfusion increases. Further observations following preoperative anemia were made in other disciplines, since severity of preoperative anemia after major abdominal surgery was additionally linked to postoperative morbidity.^32^

Transfusion rates should be kept minimal since it increases risk for overall complications, prolongs LoS and is associated with higher revision rates and mortality.^21^ But, correlation and causality remain unclear.

Effective patient blood management (PBM) was able to reduce transfusion rates, postoperative complications, LoS and mortality rates.^1^ PBM involves many elements, e.g. preoperative treatment of anemia, minimal intraoperative blood loss and patient-specific transfusion regimen. Patients aged 65 and older undergoing orthopedic or trauma surgery should be considered for transfusion with Hb below 8 g/dl, according to the german national guidelines. But transfusion threshold should be adapted to individual needs. In very old and very frail patient, a more liberal transfusion strategy could possibly reduce 90-day mortality and postoperative delirium.^3^

Delirium is a common complication, with a prevalence of 15–53 % after surgery, leading to severe complications.^25^ It is associated with longer hospital stay, accelerated cognitive decline, morbidity and mortality.^8^^,^^13^^,^^25^

Prevention of delirium is a multimodal concept, involving preoperative, perioperative and postoperative management.^29^ Modifiable risk factor for delirium, e.g. preoperative polypharmacy should be addressed and critically reevaluated.^8^ Intraoperatively, monitoring depth of anesthesia and regional anesthesia are likely to reduce postoperative delirium. Effective pain management and avoidance of benzodiazepine should be emphasized during hospital stay.^8^^,^^29^ Risk reduction seems to be the most effective strategy, with 40 % of cases being preventable, since treatment options, e.g. use of antipsychotics may reduce symptoms, but does not positively influence outcomes after delirium.^29^

When comparing to preoperative scores, functionality improved among all elderly patients after TKA.^2^^,^^11^^,^^14^^,^^17^

Multimodal and interdisciplinary concepts with specialized preoperative, perioperative and postoperative management could further improve outcomes.^16^ In Germany, the Special Orthopedic Geriatrics (SOG) care model aims to improve postoperative outcomes after elective joint arthroplasty by implementing orthogeriatric co-management (OCM), similarly used in patients with hip fractures.^9^ OCM involves e.g. early mobilization and physical therapy, as well as optimal treatment of geriatric comorbidities in a multidisciplinary team.

### 3.5 Limitations

Comparability between articles was difficult since eligible studies often use various outcomes measures. Heterogeneity and lack of data hindered further analysis. Preoperative selection and matching cohorts can create bias since patients may have not representable health status for their age. Definition of the term complication is various, resulting in different interpretations among authors. Because of the retrospective nature of the study design and data analysis, exact incidence of postoperative complications might prove challenging. The outcomes mortality and LoS ranked very low certainty of evidence, involving considerable preintervention bias due to confounding and selection of participants as well as inconsistency of results.

## 4 Conclusion

TKA can be performed safely even in patients with advanced age. Postoperative functionality improves in elderly patients. Octogenarians have longer hospital duration compared to younger patients, but there is no clear evidence for generally higher mortality or complication rates in elderly.

## CRediT authorship contribution statement

**Annemarie Rusche:** Conceptualization, Methodology, Formal analysis, Investigation, Writing – original draft, Data curation. **Georg Osterhoff:** Conceptualization, Writing – review & editing, Supervision, Project administration. **Andreas Roth:** Conceptualization, Writing – review & editing, Supervision, Project administration. **Nikolas Schopow:** Conceptualization, Validation, Writing – review & editing, Visualization, Supervision.

## Patient's consent

This research did not require patient's consent because we did a retrospective analysis of data already published.

## Ethical statement

Human Ethics and Consent to Participate declarations: not applicable.

## Funding statement

The authors received no external financial or material support for the research, authorship, and/or publication of this article.
