# Supplementary material for: Perioperative complications and postoperative outcome of primary total knee arthroplasty in octogenarians – A systematic review
Source: J Orthop. 2025 May 20;67:209–13. doi: 10.1016/j.jor.2025.05.025 (PMC12155644; doi:10.1016/j.jor.2025.05.025)
Supplement: Multimedia component 1 [file mmc1.docx]

| **Outcomes**  **N of patients**  **(studies)** | **Effect estimates**  **1** | **Certainty of the evidence (GRADE)** |
| --- | --- | --- |
| **Perioperative mortality**  **N=3,500**  **(3)** | Octogenarian Cohort vs. Younger Cohort  Not possible | Very low  Due to Risk of bias and Inconsistency |
| **Length of stay**  **N=5,547**  **(4)** | Octogenarian Cohort vs. Younger Cohort  Not possible | Very low  Due to Risk of bias and Inconsistency |

Table S1 - Summary of Findings (SoF)

The table presents mortality rates and Length of stay (LoS) after TKA. Anticipated absolute effects (95% CI) could not be provided. Calculation of relative effects was not useful, since data presented in studies is heterogeneous and no relative effects are presented. Risk of bias is very likely to appear in retrospective data. Confounding or selection of patients, possibly influencing pre-procedure bias, led to downgrading by at least one level. A general analysis of data was not possible due to heterogeneity. Absolute effect measures vary a lot throughout studies and are difficult to compare objectively. Downgrading by another level was done since chance of selective outcome measurement and reporting is given using retrospective data.
